# Perinatal stress, brain inflammation and risk of autism-Review and proposal

**DOI:** 10.1186/1471-2431-12-89

**Published:** 2012-07-02

**Authors:** Asimenia Angelidou, Shahrzad Asadi, Konstantinos-Dionysios Alysandratos, Anna Karagkouni, Stella Kourembanas, Theoharis C Theoharides

**Affiliations:** 1 Molecular Immunopharmacology and Drug Discovery Laboratory, Department of Molecular Physiology and Pharmacology, Tufts University School of Medicine and Tufts Medical Center, Boston, MA 02111, USA; 2 Department of Biochemistry, Tufts University School of Medicine and Tufts Medical Center, Boston, MA 02111, USA; 3 Department of Internal Medicine, Tufts University School of Medicine and Tufts Medical Center, Boston, MA 02111, USA; 4 Department of Psychiatry, Tufts University School of Medicine and Tufts Medical Center, Boston, MA 02111, USA; 5 Department of Pharmacy, Tufts Medical Center, Boston, MA 02111, USA; 6 Allergy Clinical Research Center, Allergy Section, Attikon General Hospital, Medical School, Athens 12462, Greece; 7 Division of Newborn Medicine, Children’s Hospital Boston, Harvard Medical School, Boston, MA 02115, USA; 8 Department of Pediatrics, University of Texas Southwestern, Childrens Medical Center, Dallas, TX 75235, USA; 9 Department of Internal Medicine, University of Texas Southwestern Medical Center, Dallas, TX 75390, USA

**Keywords:** Allergy, Autism, Brain, Inflammation, Mast cells, Prematurity, Stress

## Abstract

**Background:**

Autism Spectrum Disorders (ASD) are neurodevelopmental disorders characterized by varying deficits in social interactions, communication, and learning, as well as stereotypic behaviors. Despite the significant increase in ASD, there are few if any clues for its pathogenesis, hampering early detection or treatment. Premature babies are also more vulnerable to infections and inflammation leading to neurodevelopmental problems and higher risk of developing ASD. Many autism “susceptibility” genes have been identified, but “environmental” factors appear to play a significant role. Increasing evidence suggests that there are different ASD endophenotypes.

**Discussion:**

We review relevant literature suggesting *in utero* inflammation can lead to preterm labor, while insufficient development of the gut-blood–brain barriers could permit exposure to potential neurotoxins. This risk apparently may increase in parents with “allergic” or autoimmune problems during gestation, or if they had been exposed to stressors. The presence of circulating auto-antibodies against fetal brain proteins in mothers is associated with higher risk of autism and suggests disruption of the blood–brain-barrier (BBB). A number of papers have reported increased brain expression or cerebrospinal fluid (CSF) levels of pro-inflammatory cytokines, especially TNF, which is preformed in mast cells. Recent evidence also indicates increased serum levels of the pro-inflammatory mast cell trigger neurotensin (NT), and of extracellular mitochondrial DNA (mtDNA), which is immunogenic. Gene mutations of phosphatase and tensin homolog (PTEN), the negative regulator of the mammalian target of rapamycin (mTOR), have been linked to higher risk of autism, but also to increased proliferation and function of mast cells.

**Summary:**

Premature birth and susceptibility genes may make infants more vulnerable to allergic, environmental, infectious, or stress-related triggers that could stimulate mast cell release of pro-inflammatory and neurotoxic molecules, thus contributing to brain inflammation and ASD pathogenesis, at least in an endophenotype of ASD patients.

## Review

### Background

**Figure 1:**
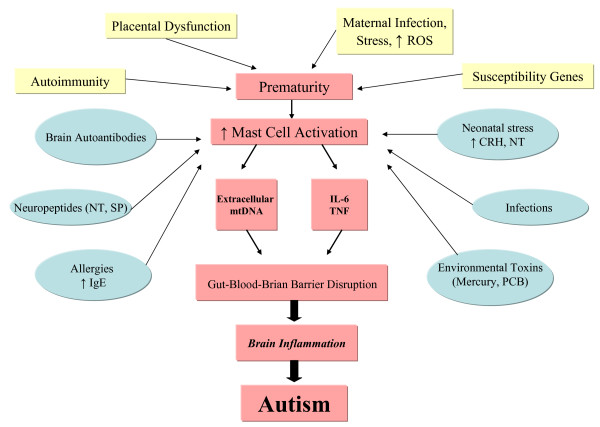
**Diagrammatic representation of proposed events and interactions during the perinatal period that may contribute to autism.** Placental dysfunction, as well as autoimmunity, maternal infection and gestational stress lead to prematurity. Defective neuronal development and susceptibility genes make the infant vulnerable to environmental triggers that activate mast cells to release mediators that disrupt the gut-blood–brain barriers causing brain inflammation. *CRH, corticotropin-releasing hormone; IgE, immunoglobulin E; IL, interleukin; LPS, lipopolysaccharide; MCP-1, macrophage chemo-attractant protein-1; mtDNA, mitochondrial DNA; NT, neurotensin; PCB, polychlorinated biphenyl; ROS, reactive oxygen species; SP, substance P; TNF, tumor necrosis factor*.

Autism Spectrum Disorders (ASD) are pervasive neurodevelopmental disorders that include autistic disorder, Asperger’s disorder, and Pervasive Developmental Disorder-Not Otherwise Specified (PDD-NOS) [1,2]. ASD are characterized by variable deficits in communication and social skills, a wide range of behavioral and learning problems and stereotypic behaviors. ASD manifest during early childhood and at least 30% of cases present with sudden clinical regression of development around 3 years of age often after acute episodes, such as a viral infection or following a vaccination [3,4]. Over the last 20 years, there has been an impressive increase in ASD prevalence of about 15% per year with current estimates of 0.5-1% of children [5,6]. A study from South Korea reported even higher rates in undiagnosed school children with ASD-like behaviors [7]. A recent report from the US Centers for Disease Control estimated that 1/88 children may be affected by ASD ( http://www.cdc.gov/Features/CountingAutism/). In the majority of cases, however, the cause of ASD is unknown [8], in spite of the apparent increase in ASD prevalence [9-11]. We propose that a number of perinatal factors contribute to focal brain inflammation and thus ASD (Figure 1).

### Prematurity

The contribution of perinatal, genetic, and immune factors in ASD was reviewed [12,13]. Premature births (less than 37 weeks gestation) have been increasing and currently account for 15% of all births in the US [14]. Infants less than 28 weeks gestation are at the highest risk for long-term neurologic problems. Placental dysfunction is a major cause of prematurity, along with intra-uterine infections and auto-immunity, which may also contribute to autism in the offspring due to anoxia [15]. An additional 5-8% of deliveries are complicated by pre-eclampsia or gestational diabetes, which may lead to placental insufficiency, abnormal growth, and postnatal metabolic imbalance [16]. *In utero* inflammation or infection can lead to preterm labor and premature birth [17-19]. A retrospective study that investigated rates of autism in children born in Atlanta, GA through the Metropolitan Atlanta Developmental Disabilities Surveillance Program (1981–93) who survived to three years of age, reported that birth prior to 33 weeks gestation was associated with a two-fold higher risk of autism [20]. A prospective study of all births less than 26 weeks gestation in 1995 in the United Kingdom and Ireland also concluded that preterm children are at increased risk for ASD in middle childhood, compared with their term-born classmates [21].

### Neurodevelopmental problems due to prematurity

Infants born between 32 and 36 weeks account for a significant increase in the rate of prematurity in the recent years [22] and are also at risk for neurologic injury [23-26]. Studies evaluating neurobehavioral outcomes following preterm birth reveal a “preterm behavioral phenotype” characterized by inattention, anxiety and social interaction difficulties, and learning difficulties [27,28].

Intra-uterine inflammation [29] can also lead to fetal brain injury and is associated with long-term adverse neurodevelopmental outcomes for the exposed offspring [30], especially in premature infants [31,32]. Cerebellar hemorrhagic injury, in particular, is associated with a high prevalence of neurodevelopmental disabilities in infants surviving premature birth [33]. A recent study reported that neonatal jaundice was associated with ASD [34].

Changes in the fetal brain lead to changes in gene expression patterns into the neonatal period. In fact, the lower the intelligence quotient (IQ), the more likely a child may display an ASD behavior [35]. One study of 1129 singleton children identified through school and health record review as having an ASD by age 8 years showed that mean IQ was significantly (p < 0.05) lower in preterm compared to term children, and term-born small-for-gestational age compared to appropriate-for-gestational age infants [36]. Gestational immune activation was reported to perturb social behaviors in genetically vulnerable mice [37].

### Low birth weight and prematurity

Results from different studies strongly suggest that prematurity and/or low birth weight (LBW) increase the risk of ASD in the offspring. One prospective study assessed 91 very LBW (<1500 g) infants, who had been born preterm, at a mean age of 22 months, and found 26% of them were likely to develop autism as suggested by a positive modified checklist for autism in toddlers (M-CHAT) test [38]. Another study showed that the diagnostic prevalence of ASD in this LBW (<2000 g) preterm cohort was higher than that reported by the Centers for Disease Control and Prevention for 8-year-olds in the general US population in 2006 [39]. A recent study found a higher risk of infantile autism among children with LBW, but suggested that suboptimal birth conditions are not an independent risk factor for infantile autism that was increased for mothers older than 35 years, with foreign citizenship, and mothers who used medicine during pregnancy [40].

### Perinatal factors contributing to higher risk of ASD

The conditions leading to premature birth may be more important than prematurity per se. For instance, the increased risk of ASD related to prematurity appeared to be mostly attributed to perinatal complications that occur more commonly among preterm infants, as shown in a cohort of 164 families with autistic children in New Jersey [41]. This was confirmed in a Swedish population-based case–control study [42]. Other population-based studies suggest that suboptimal birth conditions are not independent risk factors, but rather act as clusters to increase the risk of infantile autism [40]. A case–control population- based cohort study among Swedish children (born in 1974–1993) reported that the risk of autism was associated with daily maternal smoking in early pregnancy, maternal birth outside Europe and North America, cesarean delivery, being small-for-gestational age, a 5-minute APGAR score below 7, and congenital malformations; no association was found between autism and twin birth, head circumference, maternal diabetes, or season of birth [43].

Interestingly, a cohort study of infants born in Canada (between 1990–2002) concluded that perinatal risk factors (including prenatal, obstetrical and neonatal complications) may constitute independent risk factors for development of autism, but only for those children without a genetic susceptibility, while they appear not to influence autistic outcomes among genetically susceptible children [44]. Nevertheless, a meta-analysis on risk factors for autism concluded that there is insufficient evidence to implicate individual perinatal factors in ASD because significant association may have been observed by chance after multiple testing [45]. To the other extreme end of the spectrum, one paper had reported that estimated gestation greater than 42 weeks was associated with autism, but may play less of a role in high-functioning ASD individuals than suggested in studies of autism associated with severe retardation [46].

### Obesity

Perinatal nutritional status was shown to be related to the epigenetic status in adulthood [47]. High weight gain in pregnancy has been considered an independent risk factor for ASD in the offspring [48]. This is interesting in view of the fact that obesity has been considered an inflammatory state [49] involving release of adipocytokines [50]. Leptin is higher in obese subjects [51,52] and elevated plasma leptin levels during pregnancy are indicative of placental dysfunction [53]. Elevated plasma leptin levels were reported in children with regressive autism (n = 37), compared with typically-developing controls (n = 50) [54]. Another paper reported significantly higher leptin values in 35 patients with autistic disorder aged 180 14.1 ± 5.4 years old versus controls both at baseline and after one year of follow-up [55]. Plasma levels of leptin were also significantly increased in Rett syndrome (n = 16) compared to healthy controls (n = 16), irrespective of obesity [55]. However, there is no evidence of either a direct relationship or any role in ASD pathogenesis.

In rats, neonatal leptin administration late in the phase of developmental plasticity was able to reverse the developmental programming [56]. Mast cells also express leptin and leptin receptors, a finding implicating paracrine or autocrine immunomodulatory effects of leptin on mast cells [57].

### Genetics and environmental factors

Increasing evidence suggests that there are different ASD endophenotypes [58], possibly due to the many autism “susceptibility” genes identified [59]. In certain genetic diseases, such as Fragile X syndrome or tuberous sclerosis, autistic symptoms affect approximately 40-45% of patients [60]. Similarly, in Rett syndrome, almost 50% of patients develop ASD [61]. There is strong evidence of genetic predisposition with high rates of ASD in twins [62].

Nevertheless, a recent study of identical and fraternal twin pairs with autism showed that genetic susceptibility to ASD was lower than estimates from prior twin studies of autism, with environmental factors common to twins explaining about 55% of their risk for developing autism [63]. This partial penetration may be the result of interactions between susceptibility genes and “environmental” factors [10,64]. Environmental signals can activate intracellular pathways during early development and lead to epigenetic changes in neural function [65].

A number of mutations involving the regulatory molecule mTOR [66] and its negative control molecule Pten [67] have been linked to autism. In particular, mutations affecting mTOR have been associated with Tuberous Sclerosis I & II, but also with macrocephaly and abnormal social interactions in other diseases, such as Cowden disease [67]. Activation of mTOR [68] and reduced Pten activity [69] are also associated with increased mast cell proliferation and function.

An epidemiologic study, nested within a cohort of 698 autistic children in Denmark, concluded that perinatal environmental factors and parental psychopathology act independently to increase the risk of autism [70]. Moreover, it was recently shown that use of psychotropic medications by the mother, especially in the third trimester of pregnancy, substantially increases the risk of ASD [71]. Finally, use of general anesthesia in the newborn period was recently shown to lead to neurodevelopmental problems, such as ADHD [72,73].

Environmental toxins such as mercury [74] and polychlorinated biphenyl (PCB) [75] have been implicated in developmental neurotoxicity [76] and have been associated with ASD. Both mercury and PCBs can also stimulate mast cells [77-79].

### Oxidative stress

Several studies have suggested a link between oxidative stress and the immune response [80]. Maternal infection and inflammation can lead to oxidative stress, such as increased lipid peroxidation, but more importantly to alterations in the expression of many genes associated with adverse perinatal outcomes [81]. Oxidative stress initiated by environmental factors in genetically vulnerable individuals leads to impaired methylation and neurological deficits secondary to reductions in methylation capacity [52]. One study showed increased levels of plasma malondialdehyde, a marker of oxidative stress, in the blood of mothers who delivered preterm and in the cord blood of their preterm neonates, compared to the levels in samples from term deliveries [82]. Preterm birth was associated with increased generation of reactive oxygen species (ROS) [83]. In fact, a recent study identified an increase in the oxidative stress marker non-protein bound iron (NPBI) in the cord blood of 168 preterm newborns of gestational age 24–32 weeks [84].

A strong association between oxidative stress and autoimmunity was shown in a group of 44 Egyptian autistic children, almost 89% of whom had elevated plasma F2- isoprostane (a marker of lipid peroxidation) and/or reduced glutathione peroxidase (an anti- oxidant enzyme), compared to 44 age-matched controls [85]. Several groups have hypothesized that oxidative stress is the mechanism by which perinatal lipopolysaccharide (LPS) affects neurodevelopment in the offspring [86,87].

Brain region-specific increase in the oxidative stress markers, 3-nitrotyrosine (3-NT) and neurotrophin-3 (NT-3), especially in the cerebellum, were reported in ASD [88,89]. Another study evaluating the metabolic status of 55 children with ASD compared to 44 typically-developing children matched for age and sex reported decreased plasma levels of reduced glutathione and increased levels of oxidized glutathione, as well as low levels of S-adenosyl methionine, both major innate antioxidants [90]. Deficiencies in anti-oxidant enzymes might, in certain cases, be associated with mercury toxicity, which was shown to be tightly bound to and inactivate human thioredoxin [91]. In fact, cytosolic and mitochondrial redox imbalance was found in lymphoblastoid cells of ASD children compared to controls, an event exaggerated by exposure to thimerosal [92].

### Psychological stress

A higher incidence of stressors at 21–32 weeks gestation, the embryological age at which pathological cerebellar changes are also seen in autism, was associated with offspring developing autism [93]. Postnatal stressors in the first 6 months of life, such as death of relatives, were associated with increased risk of ASD [94]. Variations in early maternal care could affect behavioral responses in the offspring by altering at least the methylation status of the glucocorticoid receptor gene promoter [95]. Maternal stress due to the first child developing autism may also contribute to children born within a year from this first child having a much higher ASD risk [96]. ASD patients have high anxiety levels and are unable to handle stress appropriately [97]. High evening cortisol levels positively correlated to daily stressors in children with autism [98]. Moreover, increase in age of autistic children correlated with increased cortisol levels during social interaction stress [99].

Stress typically results in secretion of corticotropin-releasing hormone (CRH) from the hypothalamus and regulates the hypothalamic-pituitary-adrenal (HPA) axis [100]. Increased plasma levels of CRH have been linked to preterm labor [102,103]. CRH not only was increased in the serum of mothers who delivered preterm babies [101,103], but also correlated with the mother’s level of anxiety during that period of pregnancy [104]. Maternal serum CRH can cross the placenta, and potentially high amounts of CRH could be produced by the placenta itself, in response to external or intrauterine stress [105,106]. CRH may have an immunomodulatory role as an autocrine/paracrine mediator of inflammation during reproduction [107]. A number of cytokines, including IL-1 and IL-6, can trigger secretion of CRH from human cultured placental trophoblasts [108]. In turn, CRH stimulates IL-6 release from human peripheral blood mononuclear cells that infiltrate the fetal membranes and the placenta during intrauterine infection [109].

Acute stress also leads to high serum IL-6 that is mast cell-dependent [110]. Mast cell-derived cytokines, such as IL-6, can increase BBB permeability [110,111]. These effects may be related to the apparent compromise of the BBB in ASD patients, as indicated by the presence of circulating auto-antibodies against brain peptides [112-116]. Even though no studies have so far investigated the integrity of BBB in ASD, BBB disruption appears to *precede* any pathological or clinical symptoms associated with other brain inflammatory diseases, such as multiple sclerosis [117-119].

Mast cells have been implicated in inflammatory conditions that worsen by stress [120] and in regulating BBB permeability [110]. BBB disruption due to stress is dependent on both CRH [121] and mast cells [122]. CRH also increases intestinal permeability of human colonic biopsies [123], and has been associated with intestinal inflammation [124]. One of the early effects of immune CRH is the activation of mast cells and the release of several pro-inflammatory cytokines [125]. Increased circulating CRH, alone or with other molecules, could disrupt the gut-blood–brain barriers directly or through immune cell activation [126] and permit neurotoxic molecules to enter the brain and result in brain inflammation [127], thus contributing to ASD pathogenesis (Figure 1).

CRH can also be secreted from immune cells [128], mast cells [129], skin [130,131] and post-ganglionic nerve endings [132], leading to pro-inflammatory effects [133,134]. Moreover, CRH released from hair follicles can trigger proliferation and maturation of mast cell progenitors [135]. These findings may help explain why many children with ASD report “allergic-like” symptoms often in the absence of sensitivity to typical allergens [136] that implies mast cell activation [137].

### Maternal autoimmune diseases

 The relationship between ASD and familial auto-immunity has long been recognized [138] and has been supported by at least three large population-based studies that utilized medical records and physician data. One case–control study, nested within a cohort of infants born in California (between 1995–1999), examined the association of “immune-related conditions” and reported that maternal psoriasis, asthma, hay fever and atopic dermatitis during the second trimester of pregnancy correlated with over two-fold increased risk of ASD in their children [139]. A study of a large pediatric population (n = 689,196, born in Denmark between 1993–2004), in which 3,325 children were diagnosed with ASD including 1,089 cases of infantile autism, confirmed an association between family history of type 1 diabetes, rheumatoid arthritis, as well as maternal celiac disease and ASD [140]. A significant association between parental rheumatic fever and ASD, as well as several significant correlations between maternal auto-immune diseases and ASD, were also reported in case–control studies (n = 1,227 ASD cases) based on 3 Swedish registries [141]. A preliminary report also indicated that mothers with mastocytosis, characterized by an increased number of activated mast cells in many organs [142,143], during pregnancy had a higher risk of delivering one or more children with ASD [144].

Auto-antibodies against brain proteins have also been reported in a number of mothers with children who developed ASD [145]. The transfer of such maternal auto-antibodies to the developing fetus during pregnancy could contribute to immune dysregulation and abnormal neurodevelopment in the offspring, possibly contributing to ASD [145-148]. One recent paper provided a different perspective. In this paper, maternal IgG reactivity to certain fetal brain proteins correlated strongly with diagnosis of autism (p = 0.0005), while reactivity to at least one or more proteins correlated strongly with a “broader” diagnosis of ASD [149].

Human studies investigating the role of perinatal infection in the pathogenesis of autism are limited, and have mostly addressed viral infections [150-152] especially rubella [151,153,154]. A nationwide study of children in Denmark (n>20,000, born 1980–2005) reported an increased risk for ASD after maternal viral infection in the first trimester of pregnancy (adjusted hazard ratio = 2.98; CI: 1.29-7.15) or maternal bacterial infection in the second trimester of pregnancy (adjusted hazard ratio = 1.42; CI: 1.08-1.87) [155]. In spite of some anecdotal reports of the presence of xenotropic murine leukemia virus-related virus (XMRV) antibodies in autistic children, a recent publication detected no such virus in blood, brain or semen samples of ASD patients or their fathers [156]. Moreover, even though XMRV was reported to be present in as many as 60% of patients with chronic fatigue syndrome [157], recent reports have suggested that these findings may be due to contamination of laboratory reagents [158]. A number of rotaviruses have been isolated from many asymptomatic neonates [159] and could contribute to ASD.

### Auto-inflammation in ASD children

Some form of autoimmunity has been suspected in ASD [85,160-162]. An endophenotype with complex immune dysfunction appears to be present both in autistic children and their non-autistic siblings [163]. As mentioned earlier, brain specific auto-antibodies are present in the plasma of many ASD individuals [112,164,165]. In a cohort of Egyptian autistic children, 54.5% had antineuronal antibodies [166]. The presence of such auto-antibodies suggests a loss of self-tolerance to neural antigens during early neurodevelopment, but their precise role in autism remains unknown [85,160-162]. In particular, a recent paper reported that about 40% of children (3.2 years old) from both the Autism Phenome Project and normotypic controls contained auto-antibodies against Macaque cerebellum Golgi neurons; there was no difference except that the children with auto-antibodies had higher scores for behavioral and emotional problems [167].

An inflammatory response in ASD is supported by a number of facts. TNF was increased almost 50 times in the cerebrospinal fluid (CSF) [168], and IL-6 gene expression was increased in the brain [169] of ASD children. CSF and microglia of ASD patients also had high levels of macrophage chemoattractant-protein-1 (MCP-1) [170], which is a potent chemo-attractant for mast cells [171]. In contrast, ASD plasma contained low levels of transforming growth factor-beta1 (TGF-β1) [172]. The clinical significance of such results is not clear given some findings from animal experiments. However, brain over-expression of TGF-β1 postnatally decreased social interaction in mice [173] but chronic brain TGF-β1 over-expression during adulthood led to opposite behavior in adult mice, a finding in agreement with reduced plasma TGF-β1 found in ASD patients. In line with the postnatal TGF-β1 expression worsening ASD-like symptoms in mice, TGF-β1 and IL-9 exacerbated excitotoxic brain lesions through mast cells in mice [174]. It is intriguing that mast cell-derived IL-9 exacerbates newborn brain toxic lesions [175], induces intestinal permeability and predisposes to oral antigen hypersensitivity in children [176]. One recent paper reported that IL-9 induces mast cell release of vascular endothelial growth factor (VEGF) [177] which also inhibits gut mast cell function [178].

Other studies have reported elevations of plasma cytokines [179,180]. However, these results have been variable and do not reflect similar changes in animal models of autism. We recently reported that neurotensin (NT) is increased in serum of young children with autism [181] and can stimulate mast cell secretion [182]. We also reported that NT can stimulate secretion of extracellular mitochondrial DNA, which was also increased in the serum of these ASD patients [183]. NT is a brain and gut peptide that contributes to gut inflammation due to acute stress [123] and also acts synergistically with CRH to increase vascular permeability [184], mostly due to the action of CRH to stimulate selective release of VEGF from mast cells [185].

This finding may add to the mitochondrial dysfunction reported in many patients with ASD [186,187]. This could relate to reduced energy production [188], decreased ability to buffer ROS [189], susceptibility to mercury neurotoxicity, and to increased TNF release [190,191] that may also be associated with extracellular mitochondrial DNA that was found to be increased in serum of young children with autism [183] and act as “autopathogen”.

Mast cells are well-known for their leading role in allergic reactions, during which they are stimulated by IgE binding to high-affinity receptors (FcϵRI), aggregation of which leads to degranulation and secretion of numerous pre-stored and newly-synthesized mediators, including IL-6 and TNF [192-197]. In addition to IgE, many substances originating in the environment, the intestine or the brain can trigger mast cell activation [137]. These include non-allergic environmental, infectious, neurohormonal and oxidative stress-related triggers, often involving release of mediators selectively, without degranulation [137,198].

### Laboratory studies

Introduction of human systemic lupus erythematosus auto-antibodies to pregnant mice led to cortical impairment in their offspring [199]. Animal studies have shown that maternal immune activation (MIA) can cause both acute and lasting changes in behavior, CNS structure and function in the offspring [200]. Early life stress due to maternal separation in rats resulted in an altered brain-gut axis and was sufficient to increase blood concentrations of pro-inflammatory cytokines after a challenge with LPS [201]. A short period of restraint [202] or maternal deprivation stress [203] also increased the severity of experimental autoimmune encephalomyelitis in rodents. Maternal separation stress and CRH contributed to a dysfunctional mucosal barrier in rodents [204].

In a poly(I:C) mouse model for MIA, co-administration of anti-IL-6 antibody or use of IL-6−/− mice prevented the social deficits and associated gene expression changes in the brain of the offspring [205]. In addition to its direct detrimental effect on the placenta and fetal brain tissue, IL-1 also induces selective release of IL-6 from mast cells [206]. IL-1 receptor antagonism after systemic end-of-gestation exposure to LPS prevented neurodevelopmental anomalies in pregnant rats [207]. Bacterial LPS activates Toll-like receptor-4 (TLR-4) on immune cells leading to synthesis and release of TNF [197], IL-1 and IL-6 [18]. Moreover, viral double-stranded RNA like poly(I:C) induces release of TNF and IL-6 without degranulation from mast cells through viral TLR-3 [208].

## Conclusions

Prematurity, low birth weight and perinatal problems may contribute to increase risk of ASD. This status and susceptible genes, especially of the mTOR and Pten pathways, may make the infant more vulnerable to mast cell activation. Mast cells are now considered important in both innate and acquired immunity [209,210], as well as in inflammation [211,212], and obesity [213]. Such processes may define at least one ASD endophenotype that may be more amenable to therapy.

We propose that perinatal mast cell activation by environmental, infectious, neurohormonal and immune triggers could adversely affect neurodevelopment, disrupt the gut-blood–brain barriers, and contribute to focal brain inflammation and ASD (Figure 1). This premise could be further tested by investigating levels of CRH, NT and mtDNA in archived mother and infant blood and comparing the levels between those cases that eventually have children that develop ASD and neurotypic children. Moreover, well-designed studies could be conducted measuring potential biomarkers and providing evidence of allergic and non-allergic mast cell activation postnatally, and particularly at times of developmental regression. Reduction of stress during gestation and infancy, decrease in brain inflammation and/or mast cell activation (especially with some natural flavonoids [214,215] such as luteolin [216,217], which was recently shown to have some benefit in ASD [218]) may prove useful in at least a subgroup of infants at high risk for developing autism.

## Abbreviations

ASD: Autism Spectrum Disorders; PDD-NOS: Disorder-Not Otherwise Specified; CRH: Corticotropin-releasing hormone; MCHAT: Modified Checklist for Autism in Toddlers; LPS: Lipopolysaccharide; BBB: Blood–brain-barrier; ROS: Reactive oxygen species; NPBI: Non-protein bound iron; 3-NT: 3-nitrotyrosine; 3NT-3: Neurotrophin; DSM IV: Diagnostic and Statistical Manual of Mental Disorders; MIA: Maternal immune activation; TLR: Toll-like receptor; TNF: Tumor necrosis factor; poly(I:C): Polyinosinic:polycytidylic acid; XMRV: Xenotropic murine leukemia virus-related virus; CSF: Cerebrospinal fluid; MCP-1: Macrophage chemo-attractant protein-1; TGF-β1: Transforming growth factor-beta1; NT: Neurotensin; VEGF: Vascular endothelial growth factor; JAK2/STAT3: Janus tyrosine kinase-2/signal transducer and activator of transcription-3.

## Competing interests

The authors declare that there is no competing interest that could be perceived as prejudicing the impartiality of the research reported.

## Authors’ contributions

All authors have read and approved the final manuscript. TCT designed and wrote most of the paper. AA, SA, and K-DA, SK researched the literature and prepared the manuscript, while S.K. reviewed it and offered comments and suggestions.

## Financial support

Aspects of the research mentioned here were funded in part by grants awarded to TCT from Safe Minds, the National Autism Association, the Autism Research Collaborative, the Autism Research Institute, as well as Theta Biomedical Consulting and Development Co., Inc. (Brookline, MA).

## Pre-publication history

The pre-publication history for this paper can be accessed here:

http://www.biomedcentral.com/1471-2431/12/89/prepub
